# Propofol detection for monitoring of intravenous anaesthesia: a review

**DOI:** 10.1007/s10877-021-00738-5

**Published:** 2021-07-02

**Authors:** David C. Ferrier, Janice Kiely, Richard Luxton

**Affiliations:** Institute of Bio-Sensing Technology, University of the West of England, Frenchay Campus, Bristol, BS16 1QY UK

**Keywords:** Propofol, 2,6-Diisopropylphenol, Real-time monitoring, Optical detection, Electrochemical detection

## Abstract

This paper presents a review of established and emerging methods for detecting and quantifying the intravenous anaesthetic propofol in solution. There is growing evidence of numerous advantages of total intravenous anaesthesia using propofol compared to conventional volatile-based anaesthesia, both in terms of patient outcomes and environmental impact. However, volatile-based anaesthesia still accounts for the vast majority of administered general anaesthetics, largely due to a lack of techniques for real-time monitoring of patient blood propofol concentration. Herein, propofol detection techniques that have been developed to date are reviewed alongside a discussion of remaining challenges.

## Introduction

Propofol (2,6-diisopropylphenol) is an intravenous drug used for the induction and maintenance of anaesthesia. It has favourable characteristics, including rapid induction and a short half-life, and consequently it has been the most commonly used intravenous anaesthetic for the last 30 years [1, 2].

Until recently, the most common practice in general anaesthesia was to use an intravenous anaesthetic, such as propofol, for the induction phase and volatile anaesthetics for the maintenance phase [3]. However, it is possible to use propofol for both the induction and maintenance phases, in a process known as total intravenous anaesthesia (TIVA). There is a growing body of evidence of the advantages of TIVA over the more conventional volatile-based anaesthesia, including: reduced short-term side-effects [3], reduced cognitive effects [4–6], the potential for improved long-term survival rates for cancer patients [7, 8], and a significantly reduced environmental impact [9–11].

Despite these numerous advantages, conventional volatile-based anaesthesia still accounts for the vast majority of administered general anaesthetics world-wide [3]. One significant obstacle to a greater exploitation of TIVA is the lack of suitable methods for the continuous, real-time monitoring of blood propofol concentration in patients undergoing anaesthesia.

This paper presents a review of solution-phase propofol detection techniques and their potential application to real-time propofol monitoring. To the authors’ knowledge this represents the first review of propofol detection. There are also researchers who have investigated monitoring propofol in exhaled breath [3, 12–14]. However, as the relationship between blood propofol concentration and exhaled breath concentration is not fully understood [15], it remains unclear whether this approach will be applicable to patient monitoring; and gaseous propofol detection techniques will not therefore be covered in this review.

## Detection methods

### Chromatography

High performance liquid chromatography (HPLC) is perhaps the most commonly reported method for the detection and quantification of propofol, and considered by many to be the ‘gold standard’ for validation purposes. HPLC may be used in conjunction with a variety of measurement techniques, with the most common being fluorometric detection.

When using fluorometric detection, the most common excitation and emission wavelengths are 276 nm and 310 nm respectively [16–19], however, the use of other wavelengths has been reported [20]. Typically, the mobile phases consist of mixtures of either methanol [16] or acetonitrile [17, 18] with water. Nishio et al. have demonstrated propofol detection using fluorometric-HPLC using only water as a mobile phase by utilising a temperature-responsive polymer as a stationary phase [19]. Fluorometric-HPLC has been demonstrated for the detection of propofol from serum and whole blood samples, with the samples being pre-treated either by precipitation of proteins using acetonitrile [16–18] or by solid phase extraction [19]. Reported limits of quantification range between 3 and 400 ng/ml [17, 18], with linear ranges typically extending up to the order of 10 μg/ml.

Another common measurement technique used in conjunction with HPLC is UV photometry. Absorbance is measured at wavelengths ranging from 210 to 280 nm [21, 22] and the mobile phases typically consist of acetonitrile mixed with either an acidic buffer [21, 23] or ammonium [22]. Reported linear regions range from the order of 10 to 100 μg/ml and limits of quantification as low as 20 ng/ml have been reported [23].

The use of electrochemical measurement in conjunction with HPLC has also been reported, although this approach appears less common. Dowrie et al. have reported electrochemical detection of propofol from human serum and plasma using a mixture of methanol and an acidic buffer for the mobile phase and a measurement potential of + 0.8 V [24]. This group reports a linear range of 0.01 to 1 μg/ml. Pissinis et al. have utilised a highly alkaline mobile phase as at a higher pH propofol will be ionised and therefore oxidation can be carried out at a lower potential (ca. + 0.1 V), resulting in reduced interference [25]. Using this method, they report a limit of detection of 5 ng/ml. A summary of HPLC-based propofol detection techniques is presented in Table 1.

**Table 1:** Summary of reported HPLC propofol detection and quantification techniques

Measurement	Mobile phase	Extraction	LoQ^a^ (ng/ml)	Range (μg/ml)	References
Fluorescent	Methanol/water	Acetonitrile	50	1–10	[16]
Fluorescent	Acetonitrile/water/trifluoroacetic acid	Acetonitrile	400	0.4–40	[17]
Fluorescent	Acetonitrile/water	Acetonitrile	3	0.05–10	[18]
Fluorescent	Water	Solid-phase	NR	0.5–10	[19]
Fluorescent	Methanol/phosphate buffer (pH 4.5)	Methanol	100	0.1–3	[20]
UV	Acetonitrile/buffer (pH 2.5)	NR	750	15–75	[21]
UV	Acetonitrile/ammonium	NR	NR	37–592	[22]
UV	Acetonitrile/buffer (pH 3)	Solid-phase	20	NR	[23]
Electrochemical	Methanol/phosphate buffer (pH 2.8)	Pentane	5	0.01–1	[24]
Electrochemical	Acetonitrile/buffer	NR	15	NR	[25]

*NR* not reported

^a^Where a limit of detection (LoD) is stated in preference to a limit of quantification (LoQ), LoQ is assumed to be three times LoD

Whilst it may be a ubiquitous technique, HPLC is not well suited to point-of-care applications due to its reliance on bulky and expensive equipment. Furthermore, HPLC offers only discrete, rather than continuous measurement. It also requires complex and time-consuming sample pre-treatment methods.

Mass spectrometry is another common technique for the detection and quantification of propofol in biological samples, in conjunction with either gas chromatography [26–28] or liquid chromatography [29–32]. As for HPLC, when analysing propofol in whole blood, serum or plasma, the propofol is extracted from the sample either by solvent [26, 27] or solid phase extraction [29, 31].

Gas chromatography-mass spectrometry (GC–MS) has been demonstrated to be capable of detecting propofol in blood samples with lower limits between 2.5 and 10 ng/ml [26, 27], with linear ranges between 0.01 and 10 μg/ml. Similar values have been reported for liquid chromatography tandem mass spectrometry (LC–MS/MS) [29, 31, 32] although Vaiano et al. have reported a detection limit of 0.1 ng/ml from whole blood using this technique [33]. A summary of mass spectrometry-based propofol detection techniques is presented in Table 2.

**Table 2:** Summary of reported mass spectrometry propofol detection and quantification techniques

Technique	Extraction	LoQ^a^ (ng/ml)	Range (μg/ml)	References
GC–MS	Chloroform–ethyl acetate	10	0.01–10	[26]
GC–MS	Heptane	7.5	0.01–5	[27]
GC–MS	Ethyl acetate	325	NR	[28]
GC–MS	Dichloromethane/ethyl acetate	5	NR	[33]
LC–MS/MS	Solid-phase	5	0.005–2	[29]
LC–MS/MS	Acetone	NR	0.02–20	[30]
LC–MS/MS	Solid-phase	NR	0.01–1.5	[31]
LC–MS/MS	Methanol–acetonitrile/solid-phase	10	0.01–10	[32]
LC–MS/MS	Dichloromethane/ethyl acetate	0.1	NR	[33]

^a^Where a limit of detection (LoD) is stated in preference to a limit of quantification (LoQ), LoQ is assumed to be three times LoD

*NR* not reported

As for HPLC, the principle disadvantages of mass spectrometry techniques are the requirement for expensive and bulky equipment and the lack of capacity for continuous monitoring. A particular drawback is the requirement for lengthy analysis and sample preparation processes, with one group reporting analysis times of 40 min and sample preparation times of 300 min [28].

### Optical techniques

**Fig. 1:**
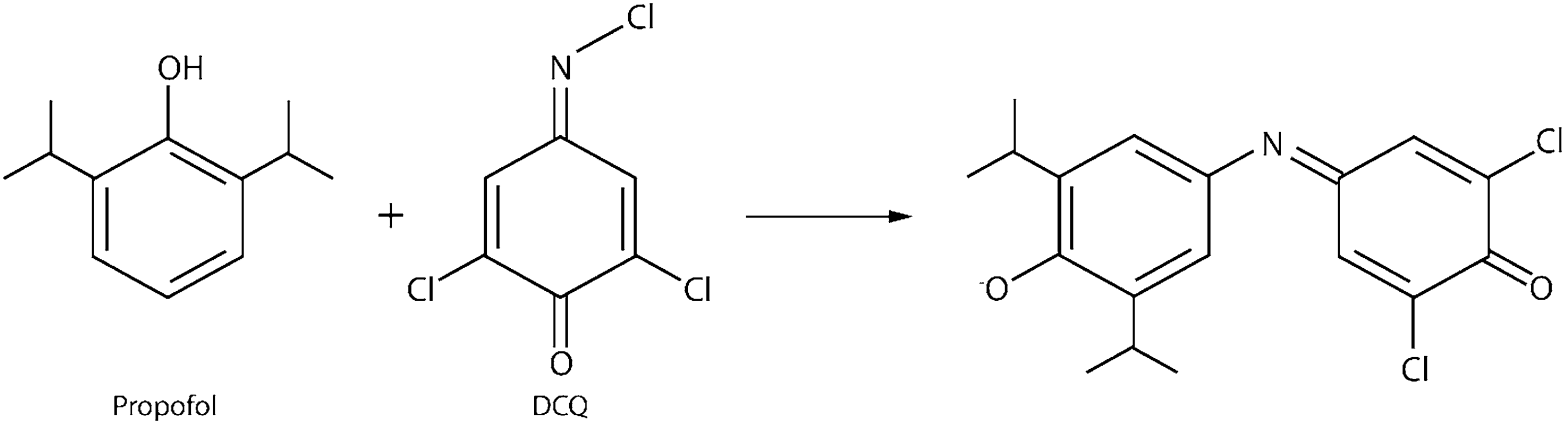
Gibbs reaction for propofol. Propofol reacts with DCQ to produce a coloured indophenol. Adapted from Mistry et al. [35]

To achieve the spectrophotometric detection of propofol, many groups have taken advantage of the Gibbs reaction. This is the name given to the process wherein 2,6-dichloroquinone-4-chlorimide (DCQ), also known as Gibbs’ reagent, reacts with phenolic compounds in alkaline conditions to produce an indophenol (Fig. 1) [34, 35]. This indophenol will be a blue-to-violet coloured species with an absorption maximum of approximately 600 nm.

Gad-Kariem and Abounassif have exploited this reaction to demonstrate the detection of propofol in biological fluids by mixing samples with DCQ solution, dimethyl sulfoxide and a buffer (pH 9.6), allowing the mixture to react for 15 min and then measuring the absorbance at 635 nm [36]. By this method this group were able to demonstrate the detection of propofol in spiked plasma and urine with a linear range of 1–5 μg/ml and a detection limit in plasma of 0.28 μg/ml.

Hong et al. have also made use of the Gibbs reaction [37]. This group have developed a disposable microfluidic chip containing a molecularly imprinted polymer (MIP) for the solid phase extraction of propofol. The MIP film is synthesised by the UV-initiated co-polymerisation of methacrylic acid and ethylene glycol dimethacrylate. The chip is used in conjunction with a laser diode and photodetector to measure the absorbance of the MIP at 655 nm, after mixing the analyte solution with DCQ within the microfluidic device. In this manner this group has demonstrated the detection of propofol in methanol solution across the range 0.25 to 10 μg/ml. It is reported that this set-up returns the propofol concentration within 60 s. However, the device cannot accommodate whole blood and this timeframe does not include the sample preparation time that would be required if working with blood, serum or plasma.

Liu et al. have also demonstrated the spectrophotometric detection of propofol by exploiting the Gibbs reaction [38]. This group have used the Pelorus 1000 system (Sphere Medical Ltd.). In this system a 0.7 ml sample of whole blood is diluted and the red blood cells lysed. Propofol is then extracted from the lysed blood by solid phase extraction and reacted with DCQ. The resultant indophenol is then detected via colourimetry. Using this system, the authors reported a limit of quantification for propofol in whole blood of 0.75 μg/ml with a linear response up to 12 μg/ml. It is reported that the time-to-results is approximately 5 min and that no sample preparation, beyond that performed automatically by the system, is required. This same system has been used to measure the blood propofol concentration of ponies under anaesthesia [39].

Sramkova et al. have demonstrated an alternative approach for the spectrophotometric detection of propofol [40]. In this method, propofol is oxidised in the presence of hydrogen peroxide in a reaction catalysed by the enzyme horseradish peroxidase (HRP). The product of this reaction is then coupled with 4-aminoantipyrine, producing a coloured solution, the absorbance of which is measured at 485 nm. By this method the authors report a propofol detection limit of 1.6 μg/ml with a linear range of 5 to 100 μg/ml. However, this approach was developed for the determination of propofol concentrations within commercial propofol emulsions, rather than within biological fluids. This same group compared the above method with a fluorometric method. By measuring the fluorescent emission of propofol at 347 nm in ethanol, they were able to detect propofol with a limit of detection of 1.3 μg/ml and a linear range of 4 to 243 μg/ml.

Li et al. have also utilised fluorescence spectroscopy for propofol detection [41]. Using an optical fibre with an on-line MIP for solid phase extraction, they have demonstrated propofol detection in whole blood with two distinct linear ranges between 0.1 and 15 μg/ml. These two linear regions are the result of two different types of binding site within the MIP. The authors report a time-to-results of 5 min.

This same group has also used a similar optical fibre to detect propofol via spectrophotometry. By reacting propofol with a diazonium salt, a coloured product can be formed with an absorption peak at 483 nm. In this manner the authors have demonstrated the detection of propofol in plasma samples across a linear range of 3 to 18 μg/ml [42]. Additionally, this group has developed a technique exploiting graphene quantum dots for the detection of propofol via fluorescence photometry. In the presence of the enzyme HRP and hydrogen peroxide, propofol will be oxidised to form 2,6-diisopropylquinone. This quinone will quench the natural fluorescence of the quantum dots, allowing for the detection of propofol to a limit of 0.5 μg/ml with a linear range of 5.34 to 89.07 μg/ml. This method was developed for the detection of propofol in emulsions [43].

El Sharkasy et al. have developed a method for the simultaneous detection of propofol and cisatracurium (a muscle relaxant commonly co-administered with propofol) by derivative synchronous spectrofluorometry [44]. By analysing the first derivative spectra at 279.6 nm, this group were able to demonstrate the detection of propofol in spiked serum samples across a linear range of 40 to 400 ng/ml with a detection limit of 4 ng/ml. No indication is given as to the reporting time of this technique, although when detecting propofol in human serum the authors employed a protein precipitation technique that requires tens of minutes to perform. A summary of reported optical propofol detection techniques is presented in Table 3.

**Table 3:** Summary of reported optical propofol detection and quantification techniques

Technique	LoQ^a^ (μg/ml)	Range (μg/ml)	References
Spectrophotometry (Gibbs)	0.84	1–5	[36]
Spectrophotometry (Gibbs/MIP)	0.75	0.25–10	[37]
Spectrophotometry (Gibbs)	0.75	0.75–12	[38]
Spectrophotometry (HRP)	5.3	5–100	[40]
Spectrophotometry (diazonium salt)	2.14	3–18	[42]
Fluorometry	4.3	4–243	[40]
Fluorometry (MIP)	NR	0.1–15	[41]
Fluorometry (quantum dots)	1.5	5.34–89.07	[43]
Derivative synchronous spectrofluorometry	0.0121	0.04–0.4	[44]

*NR* not reported

^a^Where a limit of detection (LoD) is stated in preference to a limit of quantification (LoQ), LoQ is assumed to be three times LoD

### Electrochemical techniques

It is possible to detect propofol by electrochemical techniques [24, 25, 45]. However, it is well documented that the electrochemical oxidisation of propofol will result in the deposition of an insoluble polymer film on the electrode resulting in the rapid passivation (or fouling) of the electrode surface [46–51]. The group of Lindner et al. have shown that propofol can be detected using stripping voltammetry, but that the electrode needs to be replaced or freshly polished after each measurement [45]. The same group have also reported that propofol can be detected without electrode fouling by using a restricted potential window. However, this approach results in a problematic lack of specificity. In order to address these issues, this group have developed a technique whereby electrodes are coated with a plasticised polyvinyl chloride (PVC) membrane which prevents electrode fouling, improves selectivity and lowers the limit of detection [2, 52, 53]. Propofol is highly lipophilic, and therefore it will be present in the organic membrane at a far higher concentration than an aqueous medium. Commonly interfering compounds are more hydrophilic, and will hence be less preferentially absorbed into the film. Using this technique, this group have demonstrated chronoamperometric detection of propofol using both glassy carbon and gold electrodes. They have reported a limit of detection of approximately 14 ng/ml, a linear range up to approximately 3.5 μg/ml and have demonstrated the detection of propofol in spiked human serum. One issue regarding PVC membranes is whether they possess physical and mechanical properties suitable for long-term, continuous usage [54]. This group have demonstrated these sensors for up to three hours of continuous use.

**Fig. 2:**
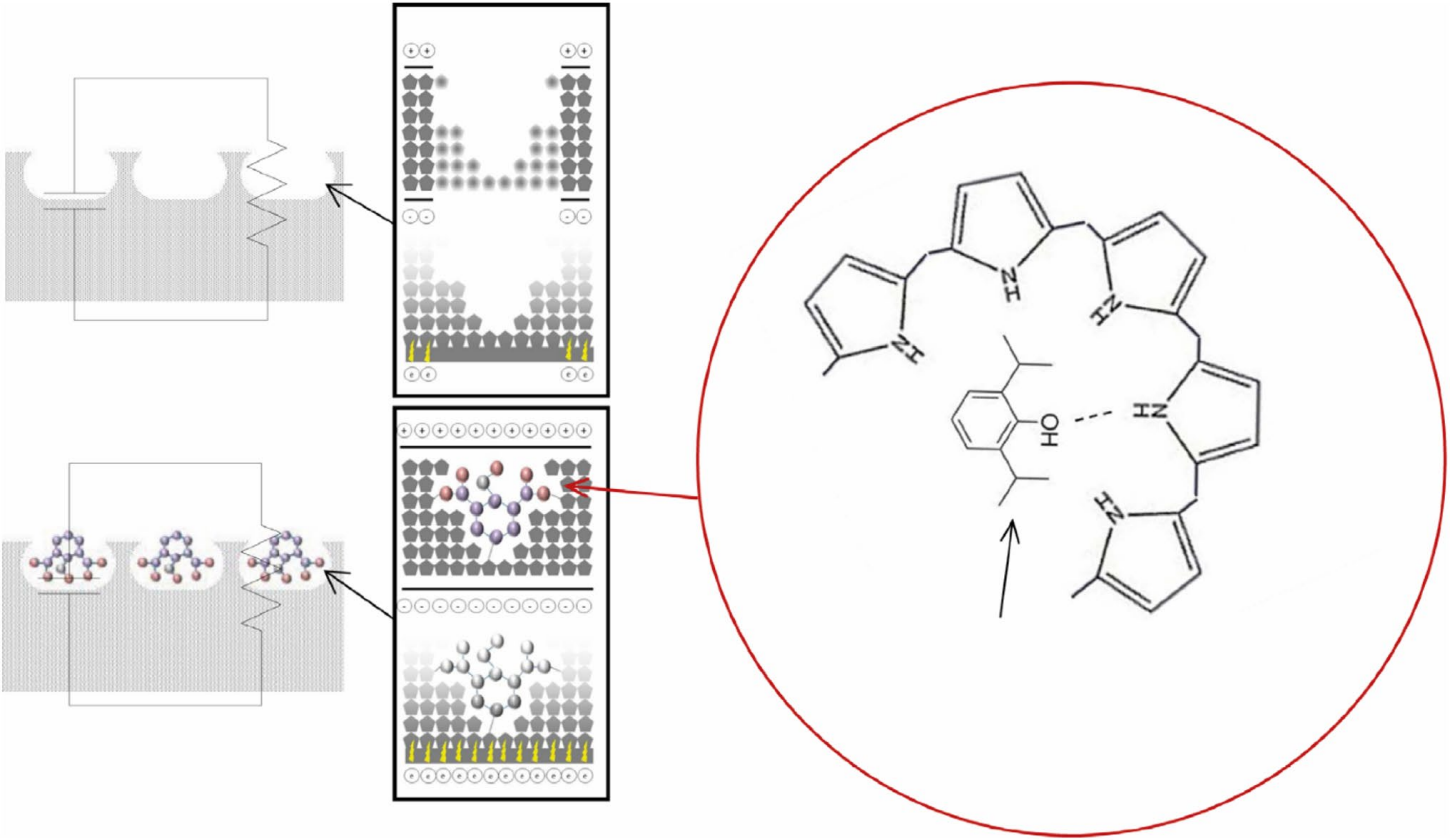
Representation of the binding of propofol molecules to a conductive molecularly imprinted polymer (MIP) and the associated equivalent circuit diagram. Reprinted with permission [55] Copyright Elsevier 2016

Hong et al. have addressed the selectivity issues by the application of a MIP [55] (this same group’s application of MIPs to optical propofol detection was outlined in the preceding section). They have developed a propofol specific MIP based on the conductive polymer polypyrrole, which is electropolymerised upon gold interdigitated electrodes. The binding of propofol to this MIP results in changes to the surface electrical properties, leading to a drop in the conductivity (Fig. 2). In this manner, this group has demonstrated a disposable biochip for chemiresistive propofol detection. The authors report a limit of detection of 0.1 μg/ml with a linear range of 0.1 to 30 μg/ml and a time-to-results of 25 s. However, continuous measurement has not yet been demonstrated. Additionally, with MIP sensors there is a possibility of slow mass transfer of the analyte to the active sites, if the pore-size distribution is heterogeneous, which is undesirable for long-term, continuous propofol monitoring [56].

Stradolini et al. have demonstrated a technique where the fouling of the electrode during propofol detection is mitigated by the inclusion of periodic electrode cleaning steps [15]. Using either boron doped diamond or pencil graphite electrodes, this group were able to demonstrate the continuous voltammetric monitoring of propofol in serum over 4 h by intermittently performing either a cyclic voltammetry-based cleaning procedure in sodium hydroxide or a chronoamperometry-based cleaning procedure in phosphate buffered saline. However, it is unclear how practical these cleaning steps would prove in the context of real-world propofol monitoring during general anaesthesia. This group reports a limit of detection of approximately 0.42 μg/ml using cyclic voltammetry. A summary of reported electrochemical propofol detection techniques is presented in Table 4.

**Table 4:** Summary of reported electrochemical propofol detection and quantification techniques

Technique	LoQ^a^ (μg/ml)	Range (μg/ml)	References
Amperometry/PVC membrane	0.043^b^	0–3.5^b^	[2]
Chemiresistive MIP	0.3	0.1–30	[55]
Voltammetry with intermittent cleaning steps	1.27^b^	NR	[15]

*NR* not reported

^a^Where a limit of detection (LoD) is stated in preference to a limit of quantification (LoQ), LoQ is assumed to be three times LoD

^b^Converted from μM

## Challenges and future prospects

The aim of delivering real-time monitoring of blood propofol concentration during general anaesthesia places a number of requirements on any potential propofol sensing technique. For instance, any method must be capable of returning results within a sufficiently narrow window of time to provide information that is of practical use to anaesthetists or other healthcare professionals. This is a major reason why approaches such as HPLC and mass spectrometry, which have a time-to-results of the order of several tens of minutes at best [28], are of limited utility for this application. Methods that require non-trivial sample pre-treatment will likely not be suitable for this same reason, and as such, sensors capable of functioning at physiological conditions will likely be more suitable than those that are not (for instance optical techniques based on the Gibbs reaction, which requires alkaline conditions [36–38]).

There are examples of groups who have reported the detection of propofol in urine as well as blood [28, 36]. However, due to the significant time-lag between administering a drug and it or its metabolites presenting in a patient’s urine, this approach will not be applicable to real-time propofol monitoring during general anaesthesia. Whole blood, serum or plasma represent the most practical biological fluids for this application.

To be of use for patient monitoring in a surgical context, any sensor system would need to be capable of producing stable results over the duration of a surgical procedure, potentially 8 h or longer. Furthermore, it has been shown that propofol will slowly redistribute between the plasma and blood cell membranes over time [57], meaning that the time between the collection and measurement of a sample will need to be tightly controlled. As such, any technique for real-time propofol monitoring must be suitable for automation, with minimal sample processing.

It is likely during general anaesthesia that propofol will be co-administered with other drugs, and as such it is necessary that any propofol sensor possess a sufficient degree of specificity. The Gibbs reagent will react with any phenolic molecule, so any optical detection technique based on the Gibbs reaction may face specificity issues if any co-administered drugs contain phenol groups. Approaches based on similar colourimetric techniques will face similar issues. Likewise, any fluorometric approach will need to ensure that there are no interfering compounds with overlapping excitation or emission windows. Specificity will also be a particular challenge for electrochemical approaches as the potential window in which propofol is electrochemically oxidised corresponds to the electroactive window for many potential interfering compounds [53].

One potential approach to improving the specificity of propofol sensors is the use of MIPs [37, 42, 55]. However, MIPs possess a finite number of binding sites and therefore may suffer from saturation effects over long timescales. Additionally, as discussed previously, there is the potential for slow mass transfer [56]. Another potential approach for improving the specificity of propofol sensors are membrane coated electrodes such as those developed by Linder et al. [2, 52, 53]. However, the performance of such electrodes over time periods of several hours has yet to be fully investigated.

When demonstrating propofol detection in plasma, serum or whole blood, the groups discussed in Sects. 2.2 and 2.3 mostly make use of spiked samples rather than real samples from patients or animals who have been administered propofol. It is known that approximately 98% of the blood concentration of propofol is bound, either to erythrocytes or serum proteins, with only the remaining 2% existing free in solution [58]; the latter free-fraction most likely being the pharmacologically active drug. Therefore, it is unclear how representative spiked samples will prove, particularly in cases such as MIPs or membrane coated electrodes where protein binding may hinder the transport of the propofol molecules to the sensor surface. Any real-time propofol detection technique intended for use with whole blood, serum or plasma will either need to be capable of detecting both bound and unbound propofol or to be capable of detecting propofol in concentration ranges up to two orders of magnitude lower than the therapeutic range (typically 0.25–10 μg/ml [52]). Few of the emerging technologies discussed in this review have sensitivities even approaching this range.

Future work in this field is likely to be focussed upon achieving the required sensitivities for the detection of the free-fraction of propofol in blood and demonstrating the required specificity to reliably differentiate propofol from potential interfering compounds. This work will be coupled with efforts to integrate such sensors with automated sample collection and processing technologies in order to achieve the real-time monitoring of blood propofol concentration that is required for the monitoring of patients undergoing general anaesthesia.

## Concluding remarks

Despite increasing evidence of the many advantages of TIVA compared to conventional volatile-based anaesthesia, both in terms of patient outcomes and environmental impact, TIVA still only accounts for a small percentage of administered general anaesthetics worldwide. The principle obstacle to a more widespread use of TIVA is the lack of suitable methods for monitoring a patient’s blood propofol concentration in real time. Existing methods such as HPLC and mass spectroscopy are too complex, expensive and slow to return results. In recent years there has been much progress in emerging propofol sensing techniques, consisting of both optical and electrochemical approaches. However, many challenges still remain, particularly in terms of sensitivity and sensor lifetime.

Electrochemical approaches are attractive due to their potential for high sensitivity and ease of automation, but possess significant challenges in terms of specificity and the potential for electrode fouling. In contrast, optical techniques generally require a greater degree of sample preparation and have not yet been demonstrated to be as suitable for continuous measurement.

Future work in this area will likely focus on improvements to sensitivity and specificity and on integrating sensors into technologies to enable automated and continuous measurement.
